# Combining molecular and landscape tools for targeting evolutionary processes in reserve design: An approach for islands

**DOI:** 10.1371/journal.pone.0200830

**Published:** 2018-07-24

**Authors:** Raquel Vasconcelos, Orly Razgour, Pedro Tarroso, Mauro Fasola, Salvador Carranza, Paulo Célio Alves

**Affiliations:** 1 CIBIO, Centro de Investigação em Biodiversidade e Recursos Genéticos, InBIO Laboratório Associado, Universidade do Porto, Campus Agrário de Vairão, Vairão, Portugal; 2 Institute of Evolutionary Biology (CSIC-UPF, Consejo Superior de Investigaciones Científicas- Universitat Pompeu Fabra), Passeig Marítim de la Barceloneta, Barcelona, Spain; 3 Biological Sciences, University of Southampton, Life Sciences Building, Southampton, United Kingdom; 4 Dipartimento Scienze della Terra e dell’Ambiente, Università degli studi di Pavia, Pavia, Italy; 5 Departamento de Biologia da Faculdade de Ciências, Universidade do Porto, Porto, Portugal; Universita degli Studi di Napoli Federico II, ITALY

## Abstract

The importance of targeting ecological and evolutionary processes in reserve design has been widely acknowledged in the literature but rarely implemented on islands. Using Socotran reptiles as models, we aim to relate richness of widespread and restricted-range species directly with landscape variables and to compare the impact of setting conservation targets for lineages versus species. Socotra Island is a UNESCO Natural World Heritage Site, containing high levels of endemism in relation to its area, especially of reptiles, the vertebrates with the most comprehensive available genetic data. We predicted the occurrences of reptile species using distribution models and used a novel approach to interpolate maps of spatial phylogenetic patterns. Patterns of intra and interspecifc diversity and differences between spatial outputs of lineage and species richness were related to eco-geographic variables. We evaluated differences in target achievement for each conservation unit within protected areas (PAs) under the current Zoning Plan (ZP) using gap and reserve design analyses. Although intraspecific richness was strongly correlated with interspecific richness, differences in their spatial distribution reached ~30% in some areas. Differences were more pronounced for wide-ranging than restricted-range taxa. Gap analysis indicates that most conservation units are under-represented in sanctuaries and that intra and interspecific richness were significantly higher outside PAs. This work will guide local-scale conservation planning as the ZP is due to be re-evaluated. This is one of the few studies on islands using genetic data from an entire class of vertebrates to incorporate lineage diversity in reserve design. This study provides an alternative methodological framework for supporting the use of landscape and genetic tools in reserve design, circumventing the use of phylogenetic distances and deterministic spatial interpolation of lineage diversity that can be widely applied to other systems.

## Introduction

In face of extensive anthropogenic land cover changes, habitat loss, climate change and accelerating extinction rates, conservation planning needs new and improved tools to prioritize the allocation of scarce resources to maximize successful conservation efforts [1]. Prioritization of areas for conservation to date has mainly focused on representing biodiversity, i. e. including targeted portion of each conservation unit within a Protected Area, rather than enabling biodiversity persistence [2]. Representativeness has been achieved by spatial analysis of quantitative distribution data to design reserve networks that offer a comprehensive representation of biodiversity features (commonly species as conservation units).

The entire range of biodiversity cannot be sampled, and much remains unknown. Therefore, better known taxa or landscape variables are commonly used in reserve design to assess how well a planning unit (i.e. conservation area or grid cell) represents the biodiversity of a wider area [3,4]. Sampling all planning units is time consuming and costly, especially in remote areas. Species distribution models (SDMs), through relating location data to environmental variables, offer an important tool for overcoming the problem of geographically sparse and spatially biased distribution data, allowing to calculate representativeness more precisely [5]. To maximize persistence, the second aim of reserve design, knowledge on genetic diversity and geophysical variables should be taken into account to preserve the evolutionary potential of populations [6]. Genetic diversity is the basis for populations to adapt to environmental changes, disease outbreaks and other extinction risks [7]. Recognizing diversity below species level and incorporating it into modelling frameworks will enable conservation planners to identify areas where populations would potentially persist [6]. Incorporating knowledge on geophysical variables is important for quantifying landscape barriers to gene flow and meta‐populations dynamics [6]. Understanding how landscape characteristics structure populations is essential for managing the genetic diversity of species and populations under threat through identifying mechanisms of isolation [8]. Contemporary landscape variables (e.g. land use) can play a role in shaping patterns of genetic diversity at fine geographic and temporal scales, while geological features and historical landscape changes (e.g. rock types) affect genetic diversity across broader spatial and temporal scales [9]. Genetic diversity acts as a reservoir for future adaptations and neutral evolution, and therefore preserving it will protect some of the processes that maintain and generate biodiversity [10]. On islands, the probability of loss of genetic diversity through drift and other neutral processes is generally higher, due to greater isolation, exposure to stochastic catasthopic events, and generally smaller population sizes [7,11]. Thus, identifying spatial patterns of genetic diversity and levels of differentiation among populations is important for guiding the assignment of conservation units and the planning of protected areas, particularly on islands [7]. However, identification of spatial patterns of intraespecific diversity is generally addressed through projecting the pairwise genetic distances between sampled individuals into mid-points between sample locations to interpolate maps of phylogenetic patterns [12]. The practice of mid-point projection is inadequate [13], because it relies on deterministic interpolation methods and assumes that pairwise genetic information is located halfway between the samples, which is unlikely to be the case especially on islands where habitat variables can vary substantially over short distances.

Genetic data can shape conservation priorities, and even predictions of protected areas (PAs) under climate change [14]. Yet, in practice, genetic diversity is often overlooked in biodiversity conservation planning [2], and reserve design studies accounting for intraspecific genetic variability in terrestrial systems are scarce, especially on island systems (e.g. [15,16]). Yet, islands host the highest levels of endemic diversity and extinction rates [11], and genetic variation is most unique, especially on large or remote islands [17]. Consequently, islands represent a large proportion of the global biodiversity hotspots [18]. Some studies have addressed included intraspecific genetic variability through using measures of phylogenetic diversity (e.g. [19]), but this approach has been questioned in case of unresolved phylogenetic relationships of taxa, and because the link between extinction probability and functional diversity, as well as with phylogenetic diversity, is controversial [20]. On islands, it is common for very closely-related radiating taxa to occupy distinct functions in the ecosystem [21], exposing the inadequacies of this framework for island systems. Globally, the distribution of intraspecific genetic diversity follows similar patterns to species richness, with increased diversity in the tropics [22], and global biodiversity hotspots offer good representation of species evolutionary history, both at the clade and species level [23]. However, at finer spatial scales, areas of high species richness are not always concordant with hotspots of genetic diversity. Therefore setting conservation priorities to maximize species representation may not be adequate for preserving genetic diversity within species [24].

**Fig 1:**
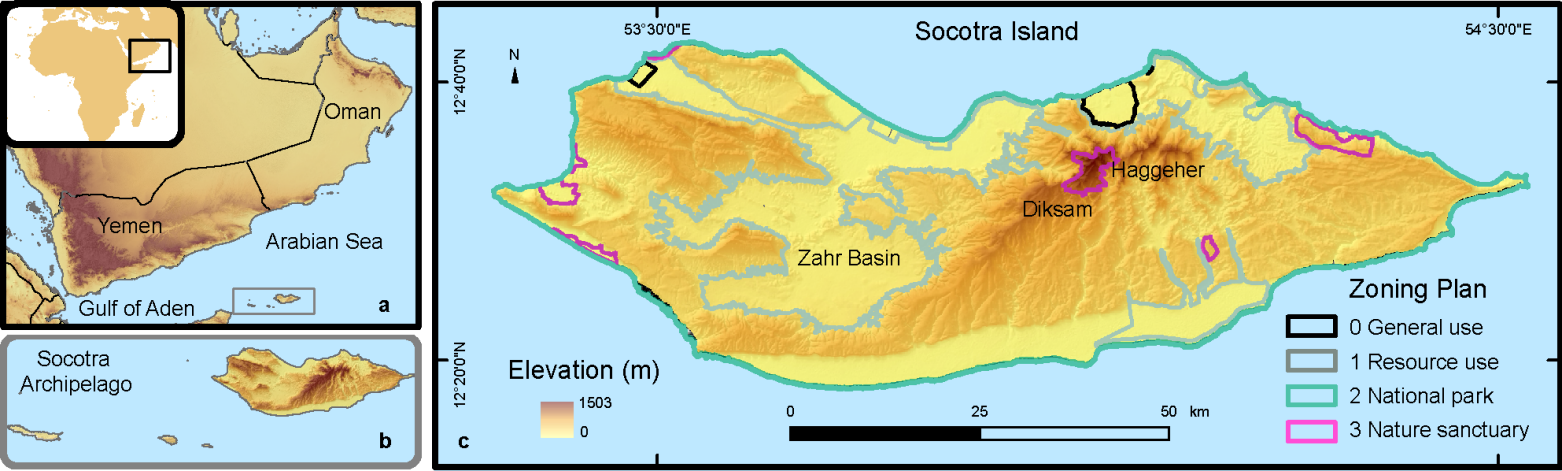
Study area. Map of the study area showing the geographic location of the Socotra Archipelago, (a, b) and the type of protection in the Zoning Plan designations over an elevation map (c). Geographic Coordinate System, Datum WGS84, Units in decimal Degrees.

The goal of the present work is to address prioritization in reserve design by combining molecular and spatial analyses circumventing the use of phylogenetic diversity and avoiding the caveats of deterministic methods in the interpolation of phylogenetic patterns. This approach was tested on the reptiles of Socotra Island, a highly suitable model system due to the characteristics of the geographic location and taxonomic group. The Socotra Archipelago (a Yemeni governorate; Socotra hereafter) is one of the most inaccessible and biodiversity rich areas worldwide [25]. It comprises four islands, of which Socotra Island is the main one, with maximum length of approximately 130 km, width of 40 km, and an area of circa 3800 km^2^ (Fig 1). Located in the Horn of Africa biodiversity hotspot, it is a UNESCO World Heritage Natural site since 2008 and considered one of the world’s most irreplaceable protected areas for conservation [26]. Yet, it is also a key under-surveyed area, with limited coverage of genetic data both in terms of data availability and taxonomic coverage [22]. Its conservation Zoning Plan (ZP) classified some areas as sanctuaries (Fig 1; 1.9% of the island’s surface) following the Biosphere Reserve Concept, and allocated other areas (26.7%) where exploitation of resources is fully or partially permitted [27]. Studies have called for the ZP to be revised to take into consideration new distribution and systematic data [28,29]. A UNEP-GEP project carried out in collaboration with the local government, has as one of its main goals “improved design and management effectiveness of the existing network of PAs”. Therefore this study will serve as the basis for selecting the best new areas to protect reptile diversity on Socotra Island. In this study we aim to: 1) identify and compare spatial patterns of species and lineage richness, determining the effect of landscape variables on these patterns; 2) assess the adequacy of the existing ZP for conserving reptile diversity; and 3) compare reserve designs based on species and lineage diversity. With this novel framework we show the importance of incorporating intraespecific diversity in reserve design.

## Methods

### Study area

Socotra Island (12°30’; N 54° E) is located in the Arabian Sea, about 250 km east from the Horn of Africa (Fig 1). Its climate is governed by the Intertropical Convergence Zone and related monsoon cycles. Its vegetation is mainly associated to arid ecosystems, with some differences between the limestone and granitic areas [30].

Socotra has a continental origin from a Gondwana fragment that split from Oman as a result of the opening of the Gulf of Aden 18 million years ago [31]. The granitic complex on Socotra Island, that includes the Haggeher mountains, was always above sea-level (Fig 1). But the limestone plateaus were formed during the Paleo-Eocene and were below sea-level during interglacial transgressions on the western side of the island [32]. Reptiles constitute the most important vertebrate fauna of Socotra. While there are no amphibians, probably only one endemic mammal, and 10 endemic bird species, all 29 native reptile species are endemic to the archipelago, 23 to Socotra Island. The reptile community of Socotra was recently barcoded (using the mitochondrial cytochrome c oxidase 1, COI, region), providing data on their intraspecific genetic diversity [28].

### Sampling

Genetic samples and distribution data of the 23 reptile species were collected during expeditions to Socotra Island between 2007 and 2014 (see Vasconcelos *et al*. 2016 [28] for further details). Using a 10x10 km square grid covering Socotra island, 103 stations (detailed in S1 Table in Supporting Information) were sampled with transects gathering 1299 genetic samples (tail tips) and 2597 presence records (one sample/record per individual). All samples used in this study were collected with appropriate permits from local authorities.

### Phylogenetic analyses

Phylogenetic analyses were carried out to determine the number of lineages present in each species based on Sanger sequencing data. Lineages were defined as a sequence or group of sequences within a species which optimized set of nodes defined the transition to inter-specific processes based on the Bayesian implementation of the General Mixed Yule Coalescent Model (bGMYC), detailed below in the lineage delimitation analyses. The datasets used for this analysis consisted of 23 alignments (one per species) of 663 base pairs of the COI gene (number of sequences per species detailed in S2 Table), downloaded from GenBank (accessions KU567304–KU567683; details in Vasconcelos *et al*. 2016 [28]). This marker was shown to detect intra-specific diversity and to have a good phylogenetic signal despite its reduced length [28]. Phylogenetic analyses could not be performed on three of the species [*Myriopholis filiformis* (Boulenger 1899), *Pachycalamus brevis* Günther 1881, and *Xerotyphlops socotranus* (Boulenger 1889); see [33] for all naming authorities] because they were represented by a single haplotype. After removing identical sequences, alignments were performed with the online application of MAFFT v.7 (http://mafft.cbrc.jp/alignment/server/). Removal of identical haplotypes is necessary because they may cause problems with downstream bGMYC analyses [34]. Best-fit models of sequence evolution were inferred in jModeltest v.2.1.3 [35] with the Akaike Information Criterion (AIC) for model selection.

An ultrametric tree was produced for each alignment using Bayesian Inference implemented in BEAST v1.8.0 [36] to feed the bGMYC analyses, because it allowed to accommodate the uncertainty associated with the estimation of a phylogeny [37]. The bGMYC R package [37] was subsequently used to determine the number of deep mitochondrial lineages per species using an R-library version 3.3 [38]. Two individual runs of 5x10^7^ generations were carried out, sampling at intervals of 10,000 generations. Preliminary tests were performed to study models and piors. Models and prior specifications applied were as follows (otherwise by default): model of sequence evolution as calculated by jModeltest [35]; constant size coalescent tree prior; uniform strict clock prior (0–100); random starting tree; base substitution prior Uniform (0,100); alpha prior Uniform (0,10) [39]. Posterior trace plots and effective sample sizes (ESS) of the runs were monitored in Tracer v1.5 [40] to ensure convergence. The results of the individual runs were combined in LogCombiner discarding 10% of the samples and the maximum clade credibility (MCC) ultrametric tree was produced with TreeAnnotator (both provided with the BEAST package). All information related to each species alignment including model selected, number of variable and parsimony-informative positions, and the results of bGMYC are presented in S2 Table.

### Lineage delimitation analysis

Deep mitochondrial lineages within Socotran reptiles were objectively identified and delimited using the General Mixed Yule-Coalescent Model implemented in a Bayesian framework (bGMYC) [41,42]. This model detects the most likely tree depth (in the single-threshold approach) at which the pattern of tree branching shifts between a Yule process (reflecting inter-specific phylogenetic structure) and a coalescent process (reflecting intraspecific structure). Because inference of lineages relies on point estimates of the topology and branch lengths, the associated phylogenetic error could decrease the accuracy of delimitation results. Therefore, we assessed uncertainty in phylogenetic tree estimation and model parameters with a Bayesian implementation of the GMYC model, which integrates over these potential sources of error via MCMC simulation [37]. We used the R-package bGMYC to calculate marginal posterior probabilities of lineage limits from the posterior distribution of ultrametric trees reconstructed with BEAST. A post-burn-in sample of 250 trees resampled from that posterior was used to calculate the posterior distribution of the GMYC model. We ran the bGMYC analysis for 100,000 generations with a burn-in of 10,000 generations.

### Spatial modelling and analysis

SDMs were generated for 20 of the 23 reptile species of Socotra Island, using Maxent v3.3.3 [43]. The initial dataset included a total of 1774 presence records and 14 landscape variables (LVs; S3 Table). Presence location records were obtained from bibliographic sources and fieldwork observations (detailed in Vasconcelos *et al*. 2016 [28]), and were mapped in ArcGIS v10 (ESRI). LVs were extracted from previous studies [30,32,44], freely available websites (http://srtm.csi.cgiar.org) or derived from other LVs (detailed in S3 Table). LVs data came from Digital Elevation Models, local weather stations, geological maps, and habitat cover data at a spatial resolution of approximately 100 m (0.0009°) to match the resolution of the location records. All habitat variables are presented as percent cover in each cell, except for linear water features (*wadis*), for which the Euclidean distance of each cell from the closest *wadi* was calculated using the ‘Euclidean Distance’ tool in ArcGIS.

A total of 10 model replicates were run with random seed which allows a different random training/testing data partition in each run. For species with <20 location records, we ran either eight (for N<20) or four (N<10) replicates. In each run, 30% of the data were set aside for model testing using bootstrapping. After testing test different values of regularization and number of features, models were run with the default settings and features. The area under the curve (AUC) of the receiver-operating characteristics (ROC) plot was used to measure model fit (good if >0.75). Standard deviation between individual model replicates was used as an indication of prediction uncertainty. The final models included eight LVs with the lowest absolute correlation scores (R≤0.75) and 657 presence records (available at figshare DOI: 10.6084/m9.figshare.4978223) after attempting to minimize the autocorrelation by removing locations that were clustered in the tridimensional LVs PCA space using the ‘near 3D’ function in ArcGIS. SDMs could not be generated for three of the 23 species (*Myriopholis filiformis*, *M*. *wilsoni* (Hahn 1978), *and Xerotyphlops socotranus*) because they had too few number of location records (≤5), therefore only grids with location records were used.

The minimum training threshold was used to transform probability of occurrences into binary maps, representing the predicted suitable range of each species. This thresholding uses all training points and is preferred when data quality is high. Moreover, when designing PAs, it is more appropriate to use a less restrictive threshold [45]. We calculated the true skill statistics (TSS) as an alterative measure of accuracy of the binary models. TSS ranges from -1 to 1 and if values are higher than 0 indicates a performance better than random [46]. To determine the suitable range of each lineage for species with more than one lineage, PHYLIN R-package [13] was used to spatially interpolate genetic distances from phylogeographic data with kriging [13], circumventing the projection of pairwise genetic distances into mid-points between sample locations [13]. The output of PHYLIN was transformed into binary data, using 0.5 probability as threshold, and intersected with the binary SDMs of the respective species to keep only areas with high probability of occurrence in lineage binary models. In total, 45 lineage suitability maps were obtained. Those, together with the species binary SDMs, comprise the dataset for 57 biodiversity features. SDM outputs were overlaid in ArcGIS to generate richness maps for all species, all lineages, as well as wide-range and restricted-range biodiversity features (that occupy less than 30% of the Socotra Island area, following recommendations in [[47]), resulting in a total of six layers of relative richness. Differences between species and lineage richness output maps (all species/lineages, wide-range and restricted-range species/lineages) were calculated in ArcGIS. Relative richness maps were reclassified into binary SDMs using as threshold the upper quartiles values (75th percentiles). To identify landscape variables associated with high levels of species/lineage diversity, the diversity values of each grid cell in the richness layers for total, wide-range and restricted-range features were directly correlated against the values of the same grid cells for the 14 available LVs, as well as latitude and longitude using the Pearson correlation coefficient (ρ) in R [38].

### Gap analysis

A gap analysis was carried out to assess how much the different categories of the ZP encompass biodiversity features and richness [48,49]. The ZP map [27] was georeferenced and the four categories were digitized into polygons. These categories present increasing levels of protection: 0 = general use, allowing a significant level of habitat modification; 1 = resource use reserve, resource use hereafter, allowing a moderate level of sustainable habitat modification; 2 = national park, allowing sustainable use of natural resources; 3 = nature sanctuaries, sanctuaries hereafter, requiring special permits to enter (Fig 1). Category 1 to 3 were considered as Protected Areas. This layer was converted into a grid with the same spatial resolution as the SDMs (~100 m) and intersected with the 57 binary SDMs of all biodiversity features in ArcGIS to calculate the number of cells that fall within each ZP category. Percent inclusion of each species/lineage suitable range within each category was calculated through dividing the number of cells within each category by the total number of grid cells in the binary SDM. This analysis was repeated for all species, lineages and restricted-range biodiversity features richness SDMs. Richness of wide-range biodiversity features was not included in the gap analysis because of their lower conservation value.

Representation targets (percentage of suitable range that should fall within PAs) were calculated according to biodiversity feature attributes, including threatened status, level of protection, and distribution ranges. Following the approach used in previous studies [50–52], Critical Endangered (CR) species should be entirely within a PA and higher proportions of representation should be achieved for restricted-range biodiversity features. A minimum target of 10% was set for the feature with the widest range and 100% for the narrowest, then targets of features with intermediate geographic ranges were log-linearly interpolated between these extremes. Only one of the study species is threatened, *Hemidactylus dracaenacolus* Rösler & Wranik 1999, and classified as Critically Endangered (CR), so a 100% target was set for this species. Four additional restricted-range species are considered Data Deficient (DD), and for those a plus of 30% of representation target was also set.

We did not consider phylogenetic diversity because most of the phylogenetic relationships of Socotran reptiles with other taxa are still unresolved. Additionally, examples of niche divergence and ecological diversification were already detected among closely-related reptiles in Socotra [53,54], suggesting a detach between branch lengths and functional diversity that would hamper a phylogenetic diversity approach. Biodiversity features were considered to be protected if percent cover within PAs was higher than the set representation target. Representation targets for relative richness of total species/lineages and restricted-range features were set as 100% of the 75th percentiles, and were considered to fail target achievement if below this threshold. Target achievement for each biodiversity feature was calculated as a percentage of the sum of the proportion of cells within PAs in respect to the representation target.

### Reserve design

Two reserve design analyses were carried out separately for species and lineages, using Zonation v4.0 [55], a software designed for spatial conservation prioritization that is frequently used in reserve design studies [56]. This software uses a gradient-like iterative heuristic to produce a sequential removal of grid cells throughout the ZP for achieving optimal solutions [55]. Target-based planning was chosen as removing rule because the goal was to find the best solution in which the maximum number of lineages met different conservation targets for both runs. In order to generate spatial aggregation within the solution, the rule ‘only remove from edges’ was selected for both runs. The Boundary Length Penalty, which devalues reserve structures with a high extent of edge, was chosen as the method for inducing reserve network aggregation, because some biodiversity features have fragmented distributions and it is one of the best methods for generating aggregation for management purposes [55]. The warp factor was set to one with an aggregation level of 0.4 based on several runs with different values. A cost layer was added to both analyses, so that grid cells with main roads and urban areas (with a buffer radius of 112 m or 1 km, respectively) were given a high cost when selected (100), secondary roads and resource use areas a middle cost (50), national parks a cost of 25 and sanctuaries a minimum cost (1).

Grid cells with highest rank have highest conservation value. The minimum set of grid cells with higher rank in the final solutions, which assured that all species or lineages were represented and achieved the targets, were selected for reclassifying the solution into binary files.

## Results

### Phylogenetic and spatial analyses

In total, we identified 45 lineages (mean = 2.0±1.3 lineages/species; range: 1–6). The species with highest number of lineages (6) was *Hemidactylus pumilio* (Boulenger 1899), while 11 of the 23 species only had one lineage (S2 Table). The number of lineages identified per species with bGMYC was always within the range of number of entities identified using different distance and tree-based techniques in Vasconcelos *et al*. (2016) [28]. Theoretical variogram models fitted heuristically to the empirical variogram obtained from PHYLIN showed the best representation of the genetic structuring in space.

SDMs performed well and had high discrimination and predictive abilities (mean AUC_train_ = 0.90±0.06 [range: 0.73–0.99], mean AUC_test_ = 0.83±0.006 [range: 0.73–0.98]) and low standard deviations (Table 1). The TSS mean values (S4 Table) are also good (mean TSS = 0.40), even for restricted-range taxa (mean TSS = 0.48). The LVs with highest contributions to all species’ SDMs were shrubland, slope and woodland (Table 1). The LVs with higher contributions to the SDM of the CR gecko *H*. *dracaenacolus* are temperature, total solar radiation and slope, and to the DD trogonophid *P*. *brevis* are distance to wadis and garrigue cover. The LVs with higher contributions to the SDMs of the remaining species are highlighted in Table 1, and LVs correlated with richness are detailed in Table 2 (see following section).

**Table 1:** Spatial modelling results. Total number of presence data of endemic reptiles of Socotra Island used in the species distribution models (SDMs), including the training (train) and test datasets, average and standard deviation (SD) of AUC for SDM replicates, and average percent contribution of each landscape variable (LV) to SDMs. LVs contributing >20% to SDMs are highlighted in bold.

		Presence data			AUC			LV contribution							
Species	Code	Total	Train	Test	Train	Test	SD	gar	mea	shr	slo	tem	trs	wad	woo
*Chamaeleo monachus*	CHmo	18	13	5	0.82	0.77	0.09	12.2	0.8	**24.8**	10.8	11.8	2.5	7.5	**29.5**
*Ditypophis vivax*	Divi	24	17	7	0.91	0.83	0.05	11.0	14.4	**21.8**	**20.0**	1.3	5.8	15.4	10.3
*Haemodracon riebeckii*	HAri	40	28	12	0.87	0.79	0.09	13.7	8.6	10.5	**20.1**	11.6	11.9	6.3	17.3
*Haemodracon trachyrhinus*	HAtr	29	21	8	0.89	0.77	0.08	4.9	11.9	**23.6**	**32.7**	9.9	0.9	8.1	8.1
*Hakaria simonyi*	Hksi	34	24	10	0.97	0.92	0.04	2.7	10.4	5.3	7.1	3.1	**51.2**	7.4	12.6
*Hemerophis socotrae*	HMso	14	10	4	0.89	0.74	0.10	3.8	11.5	9.6	**50.0**	7.7	8.8	4.5	4.0
*Hemidactylus dracaenacolus*	HEdr	7	5	2	0.99	0.98	0.01	7.8	4.0	0.1	**21.1**	**34.5**	**27.0**	4.2	1.4
*Hemidactylus granti*	HEgr	18	13	5	0.99	0.94	0.03	5.6	0.4	0.4	3.4	**82.9**	1.4	4.3	1.7
*Hemidactylus homoeolepis*	HEho	67	47	20	0.85	0.77	0.06	13.6	3.9	12.3	10.8	12.0	8.0	9.2	**30.3**
*Hemidactylus inintellectus*	HEin	31	22	9	0.90	0.77	0.06	12.3	5.4	12.5	13.4	5.6	4.0	7.6	**39.3**
*Hemidactylus pumilio*	HEpu	40	28	12	0.91	0.84	0.05	9.0	6.9	**21.5**	**28.3**	6.3	7.5	13.3	7.3
*Mesalina balfouri*	MEba	52	37	15	0.85	0.73	0.06	13.7	7.9	**28.2**	6.3	19.9	6.6	11.2	6.3
*Myriopholis filiformis* *	MYfi	1													
*Myriopholis macrura*	MYma	9	7	2	0.73	0.76	0.07	0.0	13.4	**38.0**	**44.4**	0.0	0.0	0.8	3.4
*Myriopholis wilsoni* *	MYwi	4													
*Pachycalamus brevis*	PAbr	7	5	2	0.97	0.86	0.07	**28.5**	7.0	0.0	18.3	0.0	9.7	**31.0**	5.5
*Pristurus guichardi*	PRgu	16	12	4	0.93	0.89	0.06	15.5	0.9	17.2	11.5	**32.1**	5.7	5.9	11.1
*Pristurus insignis*	PRin	52	37	15	0.85	0.80	0.06	5.4	7.1	15.8	18.7	3.0	3.0	9.7	**37.4**
*Pristurus insignoides*	PRid	20	14	6	0.99	0.99	0.00	3.8	0.6	0.3	3.7	**80.5**	5.6	5.2	0.4
*Pristurus obsti*	PRob	16	12	4	0.90	0.83	0.07	6.0	7.9	**22.7**	**39.8**	15.9	0.3	3.8	3.6
*Pristurus sokotranus*	PRso	97	68	29	0.88	0.81	0.04	3.1	6.2	18.8	7.7	8.5	17.0	8.2	**30.5**
*Trachylepis socotrana*	TRso	66	47	19	0.87	0.79	0.05	4.0	5.7	**20.6**	6.1	3.3	9.8	8.2	**42.3**
*Xerotyphlops socotranus* *	XEso	5													

*Non-modelled taxa; gar = garrigue; mea = meadow; shr = shrubland; slo = slope; temp = temperature; tsr = total solar radiation; wad = distance to wadis; woo = woodland (S3 Table).

**Table 2:** Correlation tests. Correlation values (ρ) of richness of total, wide-range and restricted-range species/lineages, and of the differences between species and lineage richness, with latitude (lat), longitude (lon) and 14 landscape variables (described in S3 Table). Absolute ρ>0.20 are highlighted in bold. All correlations were significant (P<0.05).

Richness	lat	long	alt	ept	gar	geo	mea	mix	pre	rug	shr	slo	tem	trs	wad	woo
Total species	0.02	0.02	0.07	0.18	-0.17	0.03	0.16	0.00	0.04	**-0.34**	-0.09	**-0.40**	-0.03	**0.32**	0.04	0.11
Total lineages	0.11	**0.22**	0.06	0.09	**-0.26**	-0.07	0.12	0.04	0.08	**-0.24**	0.01	**-0.29**	-0.07	**0.22**	0.07	0.17
Total difference	**-0.23**	**-0.51**	0.04	**0.26**	**0.22**	**0.28**	0.12	-0.12	-0.08	**-0.32**	**-0.27**	**-0.33**	0.08	**0.29**	-0.08	-0.14
Wide-range species	0.01	-0.01	0.03	0.19	-0.17	0.02	0.16	-0.06	-0.01	**-0.29**	-0.06	**-0.36**	0.02	**0.26**	0.10	0.07
Wide-range lineages	0.11	**0.22**	0.03	0.09	**-0.27**	-0.10	0.12	0.01	0.04	-0.19	0.04	**-0.25**	-0.03	0.17	0.11	0.15
Wide-range difference	**-0.20**	**-0.47**	0.02	**0.30**	0.13	**0.24**	0.15	-0.16	-0.11	**-0.34**	**-0.24**	**-0.37**	0.12	**0.30**	0.01	-0.13
Restricted-range species	0.04	0.11	0.16	0.03	-0.05	0.07	0.09	0.18	**0.21**	**-0.32**	-0.15	**-0.33**	**-0.22**	**0.34**	-0.19	0.19
Restricted-range lineages	0.05	0.09	0.16	0.05	-0.06	0.08	0.09	0.16	0.19	**-0.32**	-0.16	**-0.32**	**-0.20**	**0.34**	-0.18	0.18
Restricted-range difference	-0.01	0.11	0.03	-0.06	0.00	-0.03	-0.02	0.12	0.10	-0.08	0.01	-0.07	-0.11	0.06	-0.09	0.09

gar = garrigue; mea = meadow; shr = shrubland; slo = slope; temp = temperature; tsr = total solar radiation; wad = distance to wadis; woo = woodland

On average 37.0±26.5% (range: 0.7–89.2%) of Socotra Island was predicted to be suitable for the different reptile species and lineages (Table 3). The species with the smallest predicted suitable range is *H*. *dracaenacolus*, while *Mesalina balfouri* (Blanford, 1881) has the largest predicted range (Table 3).

**Table 3:** Gap analysis results. The number (nr) of grid cells of species (sp) and lineages (ln), in total, in comparison with the island area (range), and within each category of the Zoning Plan, as well as species conservation status (status), representation targets and target achievement (≥100% if above target) of the 57 biodiversity features and the fourth quantile of richnesses is showed. Species codes follow Table 1 and lineages are identified by a number. Restricted-range species and their lineages (restr.) are shaded in grey.

Biodiversity					range	target	0 = general use		1 = resource use		2 = national park		3 = sanctuaries		target achievement	
feature	sp	ln	status	total	(%)	(%)	(nr)	(%)	(nr)	(%)	(nr)	(%)	(nr)	(%)	(nr)	(%)
CHmo	1	1	NT	292535	85.3	10.3	2947	1.0	73689	25.2	210830	72.1	5069	1.7	289588	961.9
DIvi	1	0	LC	136499	39.8	15.9	1434	1.1	22567	16.5	109478	80.2	3020	2.2	135065	623.4
DIvi_1	0	1		89221	26.0	19.5	1372	1.5	13703	15.4	71175	79.8	2971	3.3	87849	504.6
DIvi_2	0	1		104923	30.6	18.1	1434	1.4	17771	16.9	83747	79.8	1971	1.9	103489	545.8
HAri	1	0	LC	276256	80.6	10.7	2834	1.0	60715	22.0	207751	75.2	4956	1.8	273422	927.0
HAri_1	0	1		201130	58.7	12.9	2834	1.4	54848	27.3	139228	69.2	4220	2.1	198296	764.1
HAri_2	0	1		209015	61.0	12.6	2445	1.2	50538	24.2	152498	73.0	3534	1.7	206570	782.8
HAtr	1	0	LC	235772	68.8	11.8	3134	1.3	76541	32.5	152539	64.7	3558	1.5	232638	838.5
HAtr_1	0	1		224772	65.6	12.1	3134	1.4	76541	34.1	141539	63.0	3558	1.6	221638	814.6
HAtr_2	0	1		70733	20.6	21.7	0	0.0	14725	20.8	53721	75.9	2287	3.2	70733	460.7
HEdr	1	1	CR	2275	0.7	100.0	0	0.0	0	0.0	1833	80.6	442	19.4	2275	100.0
HEgr	1	1	NT	4623	1.3	90.4	0	0.0	0	0.0	2908	62.9	1715	37.1	4623	110.6
HEHo	1	0	NE	276100	80.5	10.7	2915	1.1	66317	24.0	201666	73.0	5202	1.9	273185	926.4
HEHo_1	0	1		64989	19.0	22.5	2529	3.9	21287	32.8	38387	59.1	2786	4.3	62460	426.3
HEHo_2	0	1		128216	37.4	16.4	2262	1.8	19242	15.0	103189	80.5	3523	2.7	125954	599.7
HEHo_3	0	1		191269	55.8	13.3	538	0.3	46114	24.1	141186	73.8	3431	1.8	190731	751.3
HEin	1	0	LC	187232	54.6	13.4	1756	0.9	33352	17.8	148103	79.1	4021	2.1	185476	737.6
HEin_1	0	1		60720	17.7	23.2	1630	2.7	18974	31.2	38799.0	63.9	1317	2.2	59090	418.7
HEin_2	0	1		146717	42.8	15.3	1630	1.1	28482	19.4	113327	77.2	3278	2.2	145087	646.5
HEin_3	0	1		45783	13.4	26.3	0	0.0	11396	24.9	33195	72.5	1192	2.6	45783	380.1
HEin_4	0	1		84544	24.7	20.0	304	0.4	20665	24.4	62774	74.3	801	0.9	84240	498.1
HEin_5	0	1		41857	12.2	27.4	1630	3.9	12144	29.0	26813	64.1	1270	3.0	40227	351.3
HEpu	1	0	LC	184829	53.9	13.5	2849	1.5	57635	31.2	121274	65.6	3071	1.7	181980	727.9
HEpu_1	0	1		151231	44.1	15.1	2849	1.9	46171	30.5	99848	66.0	2363	1.6	148382	651.6
HEpu_2	0	1		74310	21.7	21.2	2816	3.8	22010	29.6	47913	64.5	1571	2.1	71494	453.3
HEpu_3	0	1		36042	10.5	29.2	2474	6.9	9602	26.6	22992	63.8	974	2.7	33568	319.1
HEpu_4	0	1		28345	8.3	32.4	612	2.2	10755	37.9	16334	57.6	644	2.3	27733	302.2
HEpu_5	0	1		22302	6.5	36.0	375	1.7	4835	21.7	16348	73.3	744	3.3	21927	273.4
HEpu_6	0	1		31227	9.1	31.0	0	0.0	5315	17.0	25910	83.0	2	0.0	31227	322.1
HKsi	1	0	NT	57770	16.8	23.8	1228	2.1	6738	11.7	48045	83.2	1759	3.0	56542	411.9
HKsi_1	0	1		57526	16.8	23.8	1226	2.1	6721	11.7	47820	83.1	1759	3.1	56300	411.1
HKsi_2	0	1		24716	7.2	34.4	864	3.5	5683	23.0	18071	73.1	98	0.4	23852	280.8
HMso	1	1	NT	99927	29.1	18.5	2597	2.6	62166	62.2	33695	33.7	1469	1.5	97330	526.6
MEba	1	0	LC	305669	89.2	10.0	3150	1.0	82315	26.9	214514	70.2	5690	1.9	302519	989.7
MEba_1	0	1		302942	88.4	10.1	3150	1.0	82124	27.1	211978	70.0	5690	1.9	299792	983.8
MEba_2	0	1		90147	26.3	19.4	455	0.5	14125	15.7	74563	82.7	1004	1.1	89692	512.4
MYfi *	1	1	DD	1	0.0	100.0	0	0.0	1	100.0	0	0.0	0	0.0	1	100.0
MYma	1	0	DD	150436	43.9	15.1	2024	1.3	49215	32.7	98343	65.4	854	0.6	148412	218.7
MYma_1	0	1		147860	43.1	15.2	2024	1.4	49214	33.3	95822	64.8	800	0.5	145836	647.4
MYma_2	0	1		136390	39.8	15.9	1634	1.2	39785	29.2	94117	69.0	854	0.6	134756	622.2
MYwi *	1	1	DD	5	0.0	100.0	0	0.0	0	0.0	5	100.0	0	0.0	5	100.0
PAbr	1	1	DD	9626	2.8	83.6	924	9.6	5847	60.7	2833	29.4	22	0.2	8702	108.1
PRgu	1	1	LC	86085	25.1	19.8	955	1.1	9394	10.9	73432	85.3	2304	2.7	85130	498.5
PRid	1	1	LC	4329	1.3	97.8	0	0.0	0	0.0	2647	61.1	1682	38.9	4329	102.3
PRin	1	0	LC	250861	73.2	11.3	1992	0.8	50522	20.1	193202	77.0	5145	2.1	248869	875.2
PRin_1	0	1		220379	64.3	12.2	1992	0.9	50245	22.8	163958	74.4	4184	1.9	218387	809.3
PRin_2	0	1		75194	21.9	21.1	224	0.3	15328	20.4	57633	76.6	2009	2.7	74970	472.3
PRin_3	0	1		132040	38.5	16.1	219	0.2	31207	23.6	98746	74.8	1868	1.4	131821	618.5
PRob	1	1	LC	140257	40.9	15.7	2704	1.9	60278	43.0	75177	53.6	2098	1.5	137553	626.5
PRso	1	0	LC	268762	78.4	10.9	2800	1.0	65578	24.4	194858	72.5	5526	2.1	265962	911.0
PRso_1	0	1		86988	25.4	19.7	2470	2.8	21573	24.8	59679	68.6	3266	3.8	84518	492.1
PRso_2	0	1		160917	46.9	14.6	2266	1.4	35332	22.0	119640	74.3	3679	2.3	158651	676.4
PRso_3	0	1		159275	46.5	14.7	330	0.2	47553	29.9	109545	68.8	1847	1.2	158945	681.0
TRso	1	0	LC	248971	72.6	11.4	2865	1.2	67633	27.2	173417	69.7	5056	2.0	246106	868.0
TRso_1	0	1		236304	68.9	11.8	1733	0.7	67301	28.5	163445	69.2	3825	1.6	234571	844.7
TRso_2	0	1		180283	52.6	13.7	2621	1.5	36674	20.3	136050	75.5	4938	2.7	177662	718.7
XEso *	1	1	DD	5	0.0	100.0	0	0.0	1.0	20.0	4	80.0	0	0.0	5	100.0
**total**	23	45		342865	100		3213	0.9	88383	25.8	244795	71.4	6651	1.9	339652	99.1
**average**				126967	37.0		1600	1.5	32605	25.3	90290	69.7	2472	3.4	125368	551.3
**SD**				90988	26.5		1106	1.7	25517	15.3	65611	14.5	1698	7.1	90185	260.5
**sp total**	23	0		74159	21.6	100.0	1658	2.2	17737	23.9	52989	71.5	1775	2.4	72501	97.8
**ln total**	0	45		69468	20.3	100.0	1939	2.8	19749	28.4	46027	66.3	1753	2.5	67529	97.2
**sp restr.**	10	0		10992	3.2	100.0	717	6.5	2800	25.5	5810	52.9	1665	15.1	10275	93.5
**ln restr.**	0	11		26328	7.7	100.0	1074	4.1	6143	23.3	17366	66.0	1745	6.6	25254	95.9

* Non-modelled taxa. LC, Least Concern; CR, Critically Endangered; NT, Near Threatened; NE, Not Evaluated; DD, Data Deficient.

### Spatial pattern of species and lineage diversity

**Fig 2:**
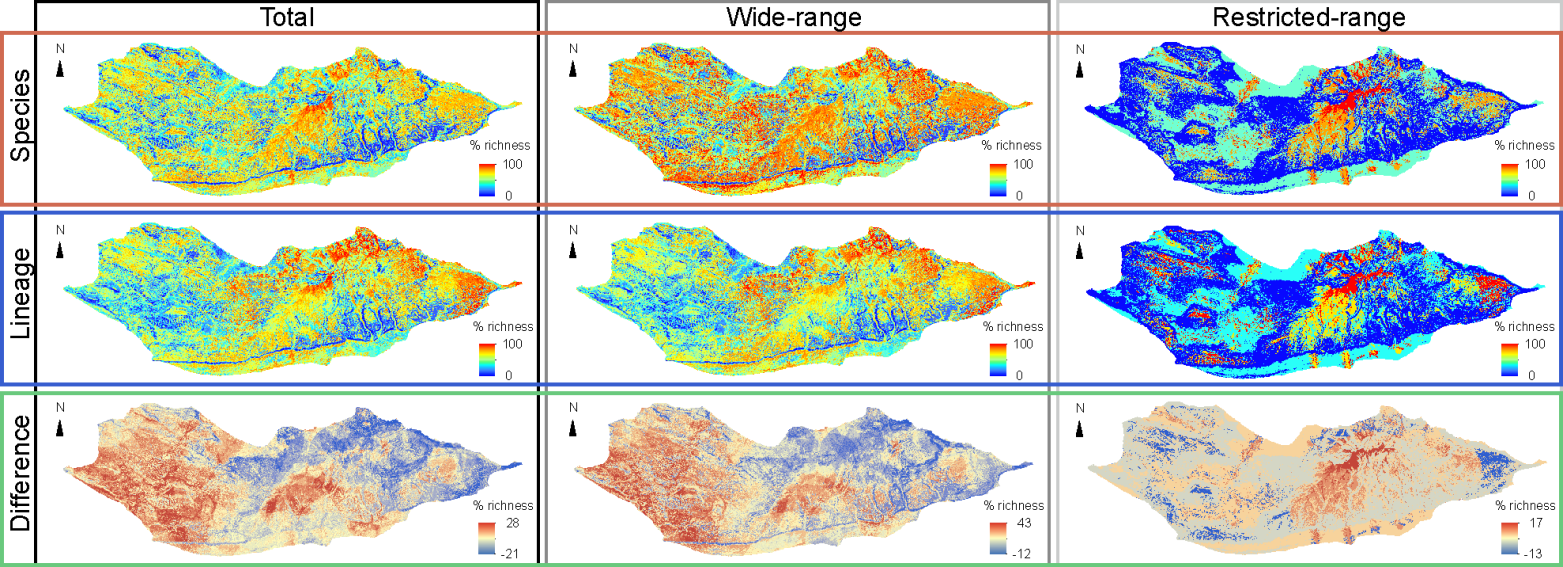
Patterns of biodiversity features. Spatial patterns of reptile diversity on Socotra Island, including maps of relative percent of species and lineage richness for the total number of biodiversity features (maximum per grid cell = 18 and 30, respectively), wide-range (maximum per grid cell = 13 and 26, respectively) and restricted-range taxa (maximum per grid cell = 5 and 6, respectively), as well as the percent differences between the species and lineage richness outputs. Geographic Coordinate System, Datum WGS84, Units in decimal Degrees.

Even though total species richness was strongly positively correlated with lineage richness (ρ = 0.93, *P*<0.0001), differences between outputs reached circa 30% in some areas (Fig 2). The area with the highest overall species richness was located in the Diksam Mountains, whilst highest levels of overall lineage richness were found in the northeast and east of the island (Fig 2). Slope was most frequently the highest correlated LV with levels of richness, followed by ruggedness (highly correlated with slope) and total solar radiation (Table 1). The richest areas for both total species and lineages were positively associated with solar radiation (ρ = 0.32 and 0.22, respectively, *P*<0.0001 for both), and negatively associated with garrigue-like habitat (basal alluvial grasslands and shrublands with *Dactyloctenium robecchii*, *Justicia rigida* and *Croton socotranus*, including mangroves; garrigue hereafter), ruggedness, and slope (ρ = -0.17 and -0.26, -0.34 and -0.24, -0.40 and -0.29, respectively, *P*<0.0001 for all; Table 1). The areas with the highest differences between species and lineage richness maps were located around the Zahr Basin, in the central plateaus, where relative species richness was considerably higher than lineage richness, and in the extreme east tip of the island, where relative lineage richness was higher (Fig 2). Differences in outputs were strongly negatively associated with longitude (ρ ^=^ -0.51, *P*<0.0001), and, unlike outputs for total species and lineages, also to shrubland (ρ = -0.27, *P*<0.0001), and positively associated with geology (ρ = 0.28, *P*<0.0001) and evapotranspiration (ρ = 0.26, *P*<0.0001; Table 1).

Spatial differences were much stronger when considering wide-range features than when considering restricted-range features (maximum of 43% versus 17%, respectively; Fig 2). While wide-range features showed similar spatial patterns as total features, restricted-range features showed the greatest differences between species and lineage richness in the Haggeher and Diksam Mountains, where relative species richness was considerably higher, and on the eastern part of the island, where the opposite pattern was evident. Restricted-range diversity (for species and lineages, respectively) was 5 to 7 folds more positively correlated with moisture and precipitation, and negatively correlated with temperature, shrubland and distance to wadis than wide-range diversity (Table 1). On the other hand, wide-range diversity was more strongly negatively correlated with garrigue than restricted-range diversity (Table 1). Differences in wide-range outputs followed the same association trends with LVs as differences between total species and lineages, while differences in restricted-range outputs were not significantly associated with either one of the LVs (Table 1).

### Gap analysis and reserve design

Almost all biodiversity features, except for one restricted-range species, *Pristurus insignoides* (Arnold 1986) and two species with higher levels of threat, the CR *H*. *dracaenacolus*, and the Near Threatened (NT) *Hemidactylus granti* (Boulenger 1899)–had very low (below 5%) representation within sanctuaries (Table 2). Twelve biodiversity features were better represented outside PAs than in sanctuaries [lineage 1 and 5 of *Hemidactylus inintellectus* (Sindaco, Ziliani, Razzetti, Pupin, Grieco 2009), lineage 1 to 3 of *Hemidactylus pumilio*, lineage 2 of *Hakaria simonyi* (Steindachner 1889), *Hemerophis socotrae* (Günther 1881), *Myriopholis macrura* (Boulenger 1899) and its lineages, *Pachycalamus brevis*, and *Pristurus obsti* (Rösler & Wranik 1999)]. One Data Deficient species, *Pachycalamus brevis*, did not reach its conservation targets within PAs.

**Fig 3:**
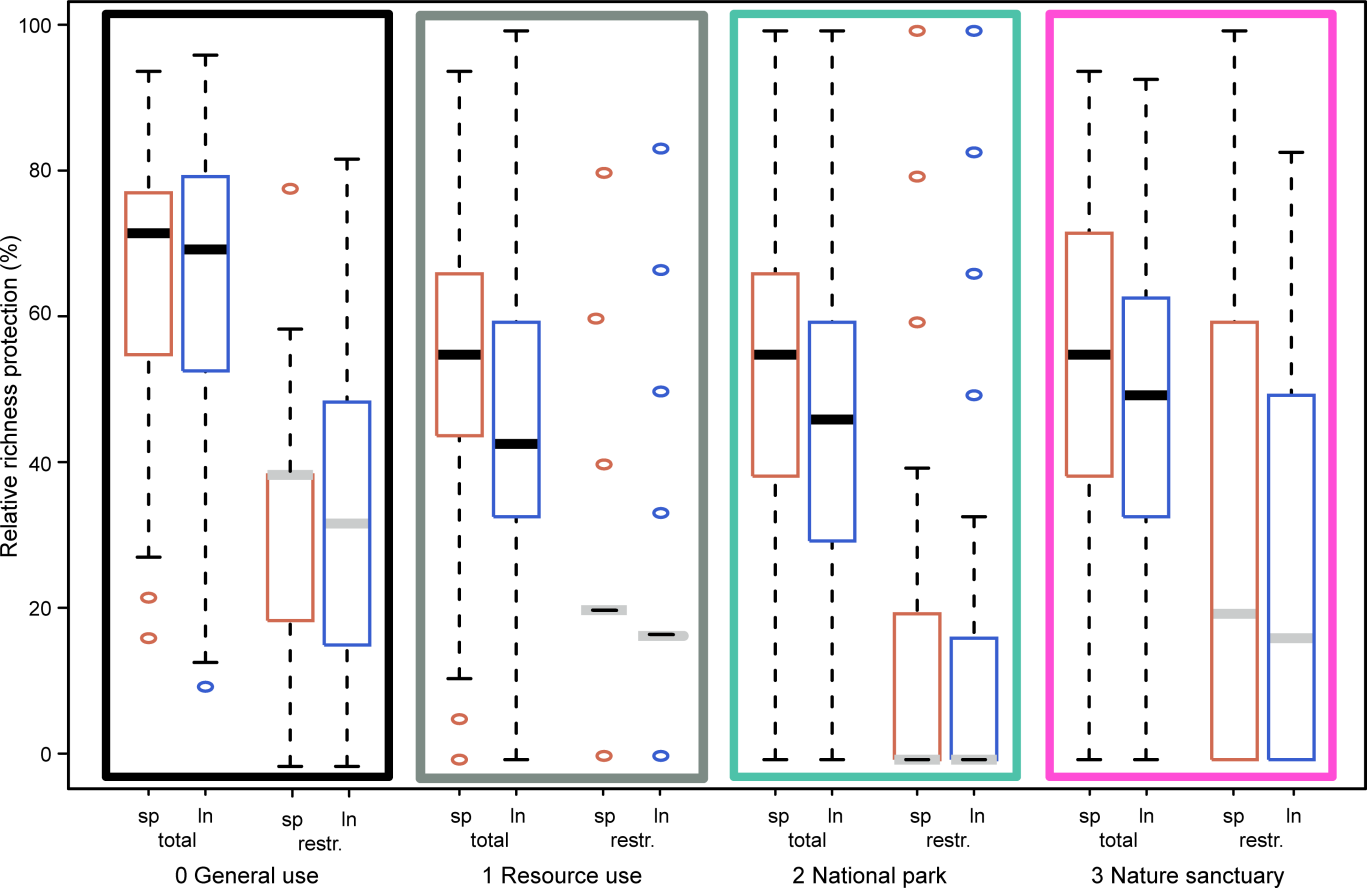
Protection of different biodiversity features comparison. Box-plots depicting the percent richness of the different biodiversity features falling within the different categories of Socotra’s Zoning Plan (rectangular colors match zoning plan category colors in Fig 1), including mean (thick line), first and third quartile (box), 1.5 interquartile range (whiskers) and outliers (dots). Categories include species richness (sp, in red) and lineage richness (ln, in blue) for total (black thick lines) and restricted-range biodiversity features (restr., grey thick lines).

For overall and restricted-range diversity, species and lineage richness was significantly higher outside than inside PAs (Generalized Linear Models: all *P-*values <0.0001; Fig 3), i.e., within category 0 (absolute richness means [95% Confidence Intervals]: species 12.17 [12.07–12.27]; lineages 19.36 [19.17–19.54]; restricted-range species 1.77 [1.73–1.81]; restricted-range lineages 2.04 [1.99–2.09]) than within categories 1 to 3 (species 9.36 [9.35–9.37]; lineage: 13.53 [13.51–13.55]; restricted-range species: 0.76 [0.75–0.76]; restricted-range lineages: 0.83 [0.82–0.83]) There was almost no difference in the representation of richness within categories 1 to 3 of the ZP (Fig 3). Nevertheless, upper quantiles are higher in sanctuaries for restricted-range species than in general use areas (Fig 3 and Table 2). Except for the latter, upper quantiles of richness representation within category 0 and 3 were similar and always below 7% of target achievement. In fact, target achievements for binary richness were all below 98% in any ZP category, and therefore did not reach assigned targets (Fig 3 and Table 2).

**Fig 4:**
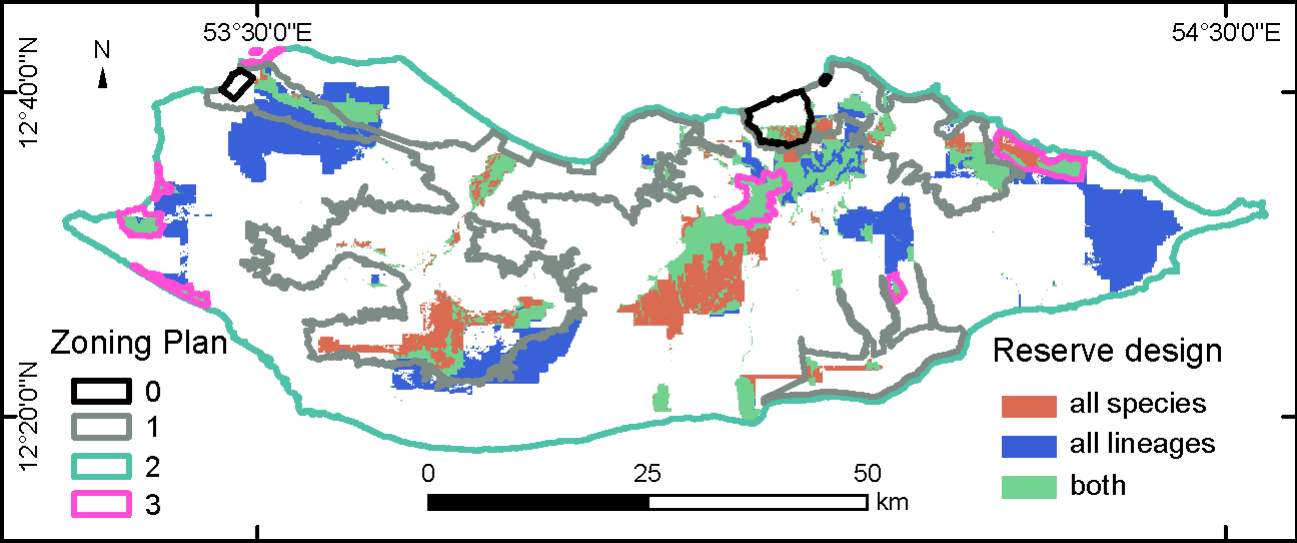
Design of new protected areas. Outputs of the reserve design analysis using either all species (red) or all lineages (blue) as targets. The areas where the two outputs coincide are marked in green. The locations of the different categories of the existing Zoning Plan are presented to highlight extent of overlap. Geographic Coordinate System, Datum WGS84, Units in decimal Degrees.

An optimal solution for designing new sanctuaries to protect a targeted proportion of all species and lineage distributions was achieved using the reserve design software (Fig 4). The binary outputs for species and lineages encompassed 12.94 and 18.62% of the island area, of which 13.22 and 8.10% were already included in sanctuaries, and 47.00 and 65.92% in national parks, respectively. On the other hand, 37.35 and 24.97%, and 2.43 and 1.03% of the species and lineages outputs intersected resource or general use areas, respectively. These values were significantly different between outputs (Chi-square = 6871.25, d.f. = 3, *P*<0.001).

## Discussion

### The importance of considering intraspecific diversity for characterizing spatial patterns

We identified and compared spatial patterns of species and lineage richness, showing that, even though total species and lineage richness are strongly positively correlated, differences between outputs reach circa 30% in some areas of Socotra Island, especially for wide-ranging biodiversity features. This highlights the importance of including lineages in conservation planning. Similarly, other studies found that conservation priorities for rainforest vertebrates in northeast Australia and large African mammals set to maximize species representation do not adequately represent patterns of intraspecific genetic diversity [14,24].

We determined the effect of environmental heterogeneity on inter and intraspecific patterns of diversity. We found that, overall, areas with highest reptile species and lineage richness are characterized by low habitat ruggedness, gentle slopes and high total solar radiation. Reptiles are ectotherms and depend on exposure to the sun for thermoregulation. In places with very steep slopes and high ruggedness sun exposure is limited. In addition, only reptiles adapted to climbing vertical surfaces could occupy those areas. Therefore observed patterns of diversity follow the expected habitat preferences of reptiles based on their ecological and physiological requirements [57]. Detailed studies on thermal and habitat ecology of the reptile species of Socotra should follow to learn more about the possible ecological and physiological drivers and constrains to intra- and interspecific diversification, similarly to what was found in other island reptiles [58]. In this study, high levels of richness are mainly associated with mountainous landscapes and areas that remained environmentally stable across geological times. Theory proclaims that species should show higher levels of genetic diversity in stable areas where suitable conditions were maintained across glaciation cycles due to long-term persistence of populations and consequent opportunities for diversification and population sub-structuring [59]. As such these refugia areas, with their higher levels of species and genetic diversity, are important for maximizing the persistence of biodiversity, particularly under global environmental change [60], and for guiding conservation planning. The pattern of increased species richness in mountain regions has been described for other islands and island taxa, including Cabo Verde plants [61], Madeiran beetles [62], and Cabo Verdean reptiles [15]. The elevational gradient on islands, associated with steep changes in climatic and habitat variables, is a strong selective pressure and an ecological barrier that promotes long-term diversification. Mountains act as islands-within-islands, with greater isolation by distance over smaller areas than lowland ecosystems, which results in higher speciation rates [61]. For example, in Socotra, the two sister taxa pairs *P*. *insignis* (Blanford 1881) and *P*. *insignoides*, and *H*. *dracaenacolus* and *Hemidactylus granti* [63] occupy different elevational ranges [34]. This phylogeographic pattern was also recovered for those species pairs when using only the COI marker [28]. The same pattern was also detected in closely-related reptile taxa in adjacent mainland Arabia [39,64].

Areas of highest lineage richness are also located in the northeast and east of the island, and largely coincide with the oldest rocks of the basement complex and Jurassic-Triassic, dated at around 476 and 145–252 million years ago (Mya), respectively [65]. Lineage richness is associated with similar landscape variables as species richness. However, lineage richness is also strongly negatively correlated with garrigue around the Zahr Basin, a shrubland habitat type found on limestone areas associated with dry climates and oceanic influence with high tolerance to salt. The distribution of this habitat on the island generally coincides with the most recent Quaternary rocks on Socotra Island that were submerged under water during interglacial periods [32]. These submersions likely resulted in population bottlenecks [66], reducing genetic diversity, that were followed by more recent population expansions, which are still observed in the star-shaped-patterns of the genetic network of most reptile species in Socotra [67,68]. Thus, older and geologically more stables areas of the island appear to have allowed the diversification and longer maintenance of lineage diversity in Socotran reptiles, including recent diversification, as seen for *Hemidactylus* [69]. Hence, the areas with the highest differences between species and lineage richness outputs were located around the Zahr Basin, where relative species richness is considerably higher than lineage richness, and in the extreme east tip of the island, where the opposite pattern is detected. Because the geological ages of areas of the island that are associated with high lineage richness, but not species richness, have a broad west-east pattern, longitude is, in turn, strongly negatively associated with differences between species and lineage richness.

Spatial differences are much stronger when considering wide-range than restricted-range biodiversity features. In general, wide-range diversity richness patterns correspond to overall diversity richness patterns, while restricted-range diversity shows a different pattern and landscape variable associations. Most LVs with important dissimilar associations with wide-range versus restricted-range diversity are water related (high moisture and precipitation, low distance to wadis and avoidance of dry shrublands). This highlights that many restricted-range species and lineages are specialists, adapted to the rare conditions of higher water availability on this arid island, as well as the importance of temperature for the diversification of reptiles [70]. Similar patterns were observed in other taxa from desert environments, such as in birds and bats [71,72].

### The effectiveness of representing intraspecific diversity

Several biodiversity features have reduced predicted suitable ranges, potentially occupying less than 10% of the island’s area, including the CR *H*. *dracaenacolus*, the NT *H*. *granti* and the DD *P*. *brevis*. This highlights the need to assess the adequacy of the existing ZP for conserving reptile diversity. Even though most biodiversity features are included within PAs, we found that additional sanctuaries (the only areas offering high levels of protection) need to be designed in order to protect a higher percentage of lineage distributions and to cover areas with highest species and lineage richness of reptiles (the main vertebrate group on Socotra). Contrary to the patterns shown in studies performed on other islands across the world [15,49,56], all individual species and lineages are well-represented within at least one of the categories of PAs in Socotra Island. Nevertheless, almost all biodiversity features have very low representation within sanctuaries, and twelve of them are better represented outside PAs than in sanctuaries. Moreover, although the suitable range of all species and lineages fall within PAs, substantial parts of the ranges of some species (e.g. *P*. *brevis*) are located partially outside national parks or sanctuary areas. Consequently, construction of infrastructures allowed in the resource use area could affect their protection in the future, especially taking into account human population growth and increasing demand for natural resources. In fact, a similar proportion of areas with the highest values of total and restricted-range biodiversity features is represented in general use areas and sanctuaries, even though the ideal situation would be for sanctuaries to encompass the richest areas, especially of restricted-range lineages. Similar to patterns observed in other parts of the world, under the existing reserve system, both average species and lineage richness is higher outside than inside PAs [73,74]. Higher richness outside PAs cannot be attributed to a low coverage of PAs on Socotra Island because this area is exceptional in that national parks and sanctuaries occupy 73% of its surface, but instead to the fact that the planning units chosen to be protected were not optimized for vertebrates.

Our reserve design study shows that using intraspecific diversity improves the results relative to the use of species diversity. Achieving targets for species requires similar number of grid cells than for lineages (12.94 versus 18.62% of the island area), however the overlap of proposed reserve areas for species with present sanctuary and natural park areas is proportionally 19% lower that with lineages, while overlap with general use areas is 2.4 fold higher. We therefore recommend the allocation of new sanctuaries for fauna, additional to the existing ones, according to our reserve design results based on lineage richness patterns (blue areas in Fig 4) because they maximize representativeness of all biodiversity features for reptiles and largely coincide with many important bird areas [29]. Lineage sanctuary areas identified in our study are generally located in close proximity to existing sanctuaries, with several new proposed areas partially falling within the boundaries of existing sanctuaries (Fig 4). Proximity to existing PAs is an important consideration because it will reduce PAs implementation and management costs, as well the renegotiation efforts with tribal chiefs of those regions. The next step will be to integrate data from other groups that could present different patterns from vertebrate taxa (e.g. invertebrates), and to use genetic markers with faster mutation rates (e.g. microsatellites) to assess connectivity (in terms of gene flow patterns) between proposed PAs.

In conclusion, we show that genetic diversity, here measured as number of lineages, represents distinct spatial patterns from the commonly used measure of species richness, which disregards evolutionary potential. Our approach to reserve design, which is based on spatial patterns of lineage diversity, enables the inclusion of the evolutionary processes of diversitification and speciation. Moreover, we highlight the importance of considering restricted-range species/lineages because their spatial patterns of richness and associations with landscape variables vary from those of wide-ranging biodiversity features and overall species/lineage richness patterns. Our proposed approach to reserve design can be readily applied to other archipelagos and terrestrial systems with available intraspecific-level genetic information.

## Supporting information

S1 Table Details on sampled stations. The codes, region name and coordinates (latitude and longitude in WGS 84) are given for each station.
(DOCX)

S2 Table Phylogenetic analyses details. Information on species codes, number of sequences (N), models of sequence evolution selected by jModelTest, number of variable, singleton and parsimony informative sites for each reptile species, and number of lineages detected using bGMYC.
(DOCX)

S3 Table Spatial modelling and analysis details. Landscape variables (LVs) used in the spatial analyses, including codes, description, average and standard deviation (SD) values, units and original source. The eight LVs with the lowest correlation scores selected for modeling the distribution of reptiles in Socotra are marked underlined.
(DOCX)

S4 Table True skill statitics values (TSS). TSS was calculated with the presences points of each species plus 10 000 random backgroud points. The selected threshold was the same as used for the SDMs (minimum trainign presence).
(DOCX)
