# Supplementary material for: Combining molecular and landscape tools for targeting evolutionary processes in reserve design: An approach for islands
Source: PLoS One. 2018 Jul 24;13(7):e0200830. doi: 10.1371/journal.pone.0200830 (PMC6057638; doi:10.1371/journal.pone.0200830)
Supplement: S1 Table — The codes, region name and coordinates (latitude and longitude in WGS 84) are given for each station. (DOCX) [file pone.0200830.s001.docx]

**Supporting Information**

**Table S1.** Details on sampled stations. The codes, region name and coordinates (latitude and longitude in WGS 84) are given for each station.

| **Station** | **Region** | **Latitude** | **Longitude** |
| --- | --- | --- | --- |
| 01_13 | Di Lishah area | 12.68638 | 54.12856 |
| 01_14 | Haggeher | 12.62606 | 54.06345 |
| 02_13 | Kariyah area | 12.63289 | 54.17030 |
| 02_14 | Haggeher | 12.59731 | 54.05370 |
| 03_13 | Kariyah area | 12.63371 | 54.21068 |
| 03_14 | Haggeher | 12.57235 | 54.04826 |
| 04_13 | NE coast (Hallah) | 12.60285 | 54.34525 |
| 04_14 | Haggeher | 12.56150 | 54.04393 |
| 05_13 | NE coast | 12.55666 | 54.44555 |
| 05_14 | Diksam | 12.54248 | 54.03875 |
| 06_13 | Ghubbah area | 12.59994 | 53.78382 |
| 06_14 | Firmihin | 12.46490 | 54.00706 |
| 07_13 | Terr Ditrur | 12.54473 | 53.71471 |
| 07A_14 | Firmihin | 12.46665 | 54.00260 |
| 07B_14 | Firmihin | 12.46040 | 54.00226 |
| 07C_14 | Firmihin | 12.46586 | 54.00113 |
| 08_13 | Shuab inland | 12.51140 | 53.43446 |
| 08A_14 | Firmihin | 12.47803 | 54.01643 |
| 08B_14 | Firmihin | 12.47570 | 54.01688 |
| 09_13 | Shuab inland | 12.51018 | 53.44876 |
| 09A_14 | Firmihin | 12.48644 | 54.00758 |
| 09B_14 | Firmihin | 12.48757 | 54.00788 |
| 10_13 | Meyhah area | 12.48979 | 53.54206 |
| 10A_14 | Shibehon | 12.48387 | 53.91278 |
| 10B_14 | Shibehon | 12.48340 | 53.91270 |
| 11_13 | Meyhah area | 12.50323 | 53.65066 |
| 11A_14 | Hallah | 12.58941 | 54.30500 |
| 11B_14 | Hallah | 12.59016 | 54.30385 |
| 12_13 | Hadibo plain | 12.63958 | 53.96387 |
| 12A_14 | Wadi Di-Fa'rhroh | 12.47449 | 54.14945 |
| 12B_14 | Wadi Di-Fa'rhroh | 12.47451 | 54.15006 |
| 13_13 | Di Rohan | 12.54595 | 53.85112 |
| 13A_14 | Killisan area | 12.53294 | 54.31505 |
| 13B_14 | Killisan area | 12.53094 | 54.31503 |
| 14_13 | Diksam | 12.51663 | 53.91137 |
| 14A_13 | Dheroh | 12.50011 | 53.98690 |
| 14B_14 | Dheroh | 12.50176 | 53.98617 |
| 15A_13 | Diksam | 12.52017 | 53.95197 |
| 15A_14 | Haggeher | 12.56453 | 54.01253 |
| 15B_13 | Diksam | 12.52126 | 53.95188 |
| 15B_14 | Haggeher | 12.56365 | 54.01158 |
| 15C_14 | Haggeher | 12.55357 | 54.00326 |
| 16_13 | Dheroh | 12.49294 | 53.99194 |
| 16_14 | Haggeher | 12.53619 | 54.07767 |
| 17_13 | Dheroh | 12.41420 | 53.94055 |
| 18_13 | East Noged | 12.38011 | 54.08138 |
| 19A_13 | Wadi Di-Fa'rhroh | 12.44134 | 54.15551 |
| 19B_13 | Wadi Di-Fa'rhroh | 12.44119 | 54.15564 |
| 20_13 | Wadi Di-Fa'rhroh | 12.49238 | 54.15072 |
|  |  |  |  |
| **Station** | **Region** | **Latitude** | **Longitude** |
| 21_13 | Go'o Area | 12.52746 | 54.17628 |
| 22_13 | Ghubbah area | 12.64930 | 53.63498 |
| 23_13 | Qalansiyah | 12.68490 | 53.49065 |
| 24_13 | Qalansiyah inland | 12.63637 | 53.57106 |
| 25A_13 | Ghubbah area | 12.65513 | 53.68183 |
| 25B_13 | Ghubbah area | 12.65536 | 53.68229 |
| 26_13 | Mori West | 12.59394 | 53.73128 |
| 27_13 | Haggher | 12.55718 | 54.13547 |
| 28_13 | Hallah | 12.55014 | 54.23694 |
| 29_13 | Timeroh area | 12.52865 | 54.29535 |
| 30A_13 | Killisan area | 12.53091 | 54.31402 |
| 30B_13 | Killisan area | 12.53159 | 54.31474 |
| 31_13 | Momi plateau | 12.53633 | 54.33745 |
| 32_13 | Momi plateau | 12.52540 | 54.39260 |
| 33_13 | Momi plateau | 12.48499 | 54.42659 |
| 34_13 | Hallah | 12.60008 | 54.21804 |
| 35_13 | Killisan area | 12.50128 | 54.33419 |
| 36_13 | Timeroh area | 12.52283 | 54.26356 |
| 37_13 | Central Noged | 12.34147 | 53.91801 |
| 38_13 | Central Noged/ Qataryiah | 12.31364 | 53.79685 |
| 39_13 | Qa'arah area | 12.33596 | 53.63230 |
| 40_13 | Qa'arah area | 12.31182 | 53.72031 |
| 41_13 | Central Noged | 12.36798 | 53.93184 |
| 42_13 | Diksam SW | 12.44204 | 53.86932 |
| 43_13 | Qabheten area | 12.41038 | 53.65985 |
| 44_13 | Qabheten area | 12.39390 | 53.62573 |
| 45_13 | Plateau W of Qabheten | 12.39466 | 53.55182 |
| 46_13 | Plateau W of Qabheten | 12.44456 | 53.50945 |
| 47_13 | Qabheten area | 12.47259 | 53.67801 |
| 48_13 | Qabheten area | 12.44578 | 53.73336 |
| 49_13 | Diksam SW | 12.42388 | 53.85360 |
| 50_13 | Diksam SW | 12.48362 | 53.91266 |
| 51_13 | NE coast | 12.56839 | 54.38943 |
| 52A_13 | Homhil | 12.57591 | 54.30545 |
| 52B_13 | Homhil | 12.57638 | 54.30719 |
| 53_13 | Di Lishah area | 12.66006 | 54.14219 |
| 54_13 | Firmihin | 12.48049 | 54.01107 |
| 55A_13 | Firmihin | 12.47575 | 54.01755 |
| 55B_13 | Firmihin | 12.47806 | 54.01656 |
| 56_13 | Firmihin | 12.46823 | 54.01175 |
| 57_13 | Rokeb area | 12.59940 | 54.16667 |
| 58_13 | Dheroh | 12.50285 | 53.98690 |
| 59_13 | Diksam | 12.51724 | 53.94100 |
| 60_13 | Robek area | 12.57375 | 54.16280 |
| AH0_13 | Meyhah area | 12.50892 | 53.58001 |
| AH1_13 | Qabheten area | 12.40380 | 53.65777 |
| AH1_14 | Haggeher | 12.61705 | 54.06064 |
| AH2_13 | Di Rohan | 12.54274 | 53.86026 |
| AH2_14 | Haggeher | 12.56528 | 54.04487 |
| AH3_13 | Wadi Ayhaft | 12.60600 | 53.99278 |
| AH3_14 | Haggeher | 12.55893 | 54.04383 |
| AH4_13 | Wadi Ayhaft | 12.61086 | 53.97878 |
| AH4_14 | Diksam | 12.52200 | 54.03600 |
| AH5_13 | Wadi Ayhaft | 12.61878 | 53.93310 |
