# Supplementary material for: Combining molecular and landscape tools for targeting evolutionary processes in reserve design: An approach for islands
Source: PLoS One. 2018 Jul 24;13(7):e0200830. doi: 10.1371/journal.pone.0200830 (PMC6057638; doi:10.1371/journal.pone.0200830)
Supplement: S2 Table — Information on species codes, number of sequences (N), models of sequence evolution selected by jModelTest, number of variable, singleton and parsimony informative sites for each reptile species, and number of lineages detected using bGMYC. (DOCX) [file pone.0200830.s002.docx]

**Supporting Information**

**S2 Table**. **Phylogenetic analyses details.** Information on species codes, number of sequences (N), models of sequence evolution selected by jModelTest, number of variable, singleton and parsimony informative sites for each reptile species, and number of lineages detected using bGMYC.

| **Species** | **Code** | **N** | **Model selected** | **Variable**  **Sites** | **Singleton**  **Sites** | **Parsimony informative** | **bGMYC lineages** |
| --- | --- | --- | --- | --- | --- | --- | --- |
| *Chamaeleo monachus* | CHmo | 8 | TPM1uf | 7 | 5 | 2 | 1 |
| *Ditypophis vivax* | Divi | 11 | TPM1uf | 17 | 6 | 11 | 2 |
| *Haemodracon riebeckii* | HAri | 14 | GTR | 78 | 3 | 75 | 2 |
| *Haemodracon trachyrhinus* | HAtr | 12 | TrN+G | 114 | 37 | 77 | 2 |
| *Hakaria simonyi* | Hksi | 6 | TPM1uf+I | 76 | 63 | 13 | 2 |
| *Hemerophis socotrae* | HMso | 3 | F81 | 3 | 3 | 0 | 1 |
| *Hemidactylus dracaenacolus* | HEdr | 10 | TrN | 18 | 2 | 16 | 1 |
| *Hemidactylus granti* | HEgr | 5 | F81 | 2 | 2 | 0 | 1 |
| *Hemidactylus homoeolepis* | HEho | 23 | GTR+G | 55 | 21 | 34 | 3 |
| *Hemidactylus inintellectus* | HEin | 52 | TrN+G | 135 | 5 | 130 | 5 |
| *Hemidactylus pumilio* | HEpu | 26 | GTR+G | 121 | 12 | 109 | 6 |
| *Mesalina balfouri* | MEba | 21 | TrN | 21 | 14 | 7 | 2 |
| *Myriopholis filiformis* | MYfi | 2 | - | - | - | - | 1 |
| *Myriopholis macrura* | MYma | 8 | HKY+I | 20 | 7 | 13 | 2 |
| *Myriopholis wilson* | MYwi | 4 | GTR | 51 | 39 | 12 | 1 |
| *Pachycalamus brevis* | PAbr | 1 | - | - | - | - | 1 |
| *Pristurus guichardi* | PRgu | 6 | HKY | 12 | 6 | 6 | 1 |
| *Pristurus insignis* | PRin | 11 | TPM1uf+G | 140 | 74 | 66 | 3 |
| *Pristurus insignoides* | PRid | 6 | HKY | 9 | 1 | 8 | 1 |
| *Pristurus obsti* | PRob | 11 | HKY | 7 | 3 | 4 | 1 |
| *Pristurus sokotranus* | PRso | 92 | TPM1uf+G | 92 | 3 | 89 | 3 |
| *Trachylepis socotrana* | TRso | 15 | HKY | 21 | 12 | 9 | 2 |
| *Xerotyphlops socotranus* | XEso | 1 | - | - | - | - | 1 |
