# Supplementary material for: Combining molecular and landscape tools for targeting evolutionary processes in reserve design: An approach for islands
Source: PLoS One. 2018 Jul 24;13(7):e0200830. doi: 10.1371/journal.pone.0200830 (PMC6057638; doi:10.1371/journal.pone.0200830)
Supplement: S3 Table — Landscape variables (LVs) used in the spatial analyses, including codes, description, average and standard deviation (SD) values, units and original source. The eight LVs with the lowest correlation scores selected for modeling the distribution of reptiles in Socotra are marked underlined. (DOCX) [file pone.0200830.s003.docx]

**Supporting Information**

| **Code** | **Description** | **Mean** | **SD** | **Unit** | **Type** | **Origin** |
| --- | --- | --- | --- | --- | --- | --- |
| alt | digital elevation model | 318.67 | 241.62 | m | topographic | [http://srtm.csi.cgiar.org](http://srtm.csi.cgiar.org/) |
| ept | evapotranspiration | 2019.75 | 171.6 | - | climatic | De Sanctis *et al.* 2013 |
| gar | garrigue-like cover | 15.41 | 27.46 | % | habitat | Malatesta *et al.* 2013 |
| geo | geological ages | - | - | - | topographic | Beydoun & Bichan 1970 |
| mea | meadow cover | 6.65 | 17.59 | % | habitat | Malatesta *et al.* 2013 |
| mix | moisture index | 0.1 | 0.05 | - | climatic | De Sanctis *et al.* 2013 |
| pre | precipitation | 194.39 | 82.54 | mm | climatic | De Sanctis *et al.* 2013 |
| rug | ruggedness index | 2.66 | 1.77 | - | topographic | derived from alt |
| shr | shrubland cover | 63.17 | 34.45 | % | habitat | Malatesta *et al.* 2013 |
| slope | slope | 8.08 | 8.68 | % | topographic | derived from alt |
| tem | temperature | 27.96 | 2.14 | ºC | climatic | De Sanctis *et al.* 2013 |
| tsr | total solar radiation | 1771.78 | 92.62 | kJ | climatic | De Sanctis *et al.* 2013 |
| wad | distance to *wadis* | 17.73 | 15.22 | % | habitat | derived from slope |
| woo | woodland cover | 3.47 | 12.94 | % | habitat | Malatesta *et al.* 2013 |

**S3 Table**. **Spatial modelling and analysis details.** Landscape variables (LVs) used in the spatial analyses, including codes, description, average and standard deviation (SD) values, units and original source. The eight LVs with the lowest correlation scores selected for modeling the distribution of reptiles in Socotra are marked underlined.
