# Supplementary material for: Combining molecular and landscape tools for targeting evolutionary processes in reserve design: An approach for islands
Source: PLoS One. 2018 Jul 24;13(7):e0200830. doi: 10.1371/journal.pone.0200830 (PMC6057638; doi:10.1371/journal.pone.0200830)
Supplement: S4 Table — TSS was calculated with the presences points of each species plus 10 000 random backgroud points. The selected threshold was the same as used for the SDMs (minimum trainign presence). (DOCX) [file pone.0200830.s004.docx]

**Supporting Information**

**S4 Table**. **True skill statistics values (TSS).** TSS was calculated with the presences points of each species plus 10 000 random background points. The selected threshold was the same as used for the SDMs (minimum training presence).

| **Taxa** | **TSS** |
| --- | --- |
| *Chamaeleo monachus* | 0.15 |
| *Ditypophis vivax* | 0.52 |
| *Haemodracon riebeckii* | 0.15 |
| *Haemodracon trachyrhinus* | 0.28 |
| *Hakaria simonyi* | 0.83 |
| *Hemerophis socotrae* | 0.56 |
| *Hemidactylus dracaenacolus* | 0.14 |
| *Hemidactylus granti* | 0.88 |
| *Hemidactylus homoeolepis* | 0.15 |
| *Hemidactylus inintellectus* | 0.42 |
| *Hemidactylus pumilio* | 0.43 |
| *Mesalina balfouri* | 0.09 |
| *Myriopholis filiformis* | - |
| *Myriopholis macrura* | 0.23 |
| *Myriopholis wilsoni* | - |
| *Pachycalamus brevis* | 0.54 |
| *Pristurus guichardi* | 0.57 |
| *Pristurus insignis* | 0.23 |
| *Pristurus insignoides* | 0.94 |
| *Pristurus obsti* | 0.53 |
| *Pristurus sokotranus* | 0.14 |
| *Trachylepis socotrana* | 0.25 |
| *Xerotyphlops socotranus* | - |
